# Advantage Management Strategy in Competition via Technological Race Perspective: Empirical Evidence from the Taiwanese Manufacturing Industry

**DOI:** 10.1155/2014/843436

**Published:** 2014-09-11

**Authors:** Tsu-Yi Hung, Yu-Ju Hsiao, Shih-Wei Wu

**Affiliations:** ^1^Department of Business Management, National Taipei University of Technology, No. 1, Sec. 3, Zhongxiao E. Road, Taipei 10608, Taiwan; ^2^Institute of Services and Technology Management, National Taipei University of Technology, No. 1, Sec. 3, Zhongxiao E. Road, Taipei 10608, Taiwan

## Abstract

This study investigated the advantage management strategies of a firm regarding the technological race in the manufacturing sector. This is to reveal whether firms adopt a catch-up or leapfrogging strategy in the competition for innovation. The results show that competition is fierce in the Taiwanese manufacturing industry. Taiwanese manufacturing firms (mostly SMEs) tend to adopt the “catch-up” strategy to keep up with their competitors in order to remain in the technological race. The result indicates that, under financial constraints, Taiwanese manufacturing firms attempt to invest in R&D to catch up with their rivals or to avoid being eliminated from the race.

## 1. Introduction

As competition intensifies, innovation becomes inevitable, thus leading to the global expansion of intellectual property rights and patent systems. Analysis of the optimal patent (the patent maximizing the dynamic social welfare of a country) by Nordhaus [1] has contributed substantially to the literature and inspired numerous scholars. Two recognizable research methods have been employed since Nordhaus [1]: the analysis of an optimal patent and the competition for innovation. Nevertheless, numerous models are based on the supposition that no firms have the required competence to realize the two types of innovation when the forerunner or leader systematically executes both types. In the competition for innovation, firms continually increase innovation to raise their competitive power. The primary consideration of technological competition is to acquire a larger market share or enhanced turnovers, which firms can achieve through “catch-up” or “leapfrogging” strategies. However, regarding the technological race, few studies have discussed these strategies. Furthermore, past studies used to adopt auction game, but it is certainly simplistic and several models have been proposed to reevaluate numerous assumptions that have reduced the empirical significance of this modeling.

This study investigated the advantage management strategies of a firm regarding the technological race in the manufacturing sector; that is, this study tried to reveal whether firms adopt a catch-up or leapfrogging strategy in the competition for innovation. The remainder of this paper is organized as follows.  Section 2 reviews the literature on the advantage management strategies of firms.  Section 3 describes the data and modeling procedures for investigating the advantage management strategies of firms.  Section 4 presents the empirical results. Finally, Section 5 summarizes our conclusions.

## 2. Literature Review

### 2.1. Competition

#### 2.1.1. Catch-Up

Catch-up is a major strategy for achieving success in an intensively competitive environment. Literature on catch-up mainly focuses on the internal development of new technologies when these technologies are emerging or the imitation and transfer of ripe technologies from multinational enterprises [2]. Denicolò and Zanchettin [3] indicated that an increase in product market competition could decrease both prospective and current profits and yet increase the difference. An intense competition stimulates R&D investment if, in a catch-up type industry, competitors have access to the same technology [4]. Aghion et al. [5] showed that catch-up rivalry promotes growth. Frontier technology advances twice as fast in the catch-up industry as in any other type of industry. Increased competition in the product market can stimulate R&D by increasing incremental profits from innovation. However, a relaxation of patent protection that reduces the R&D effort of a firm with any given lead size might still increase the economic growth rate by forcing more firms into the catch-up state in which they are induced to spend more on R&D [5].

Perez and Soete [6] indicated that a real catch-up process could be achieved only through acquiring the capacity for participating in generating and improving technologies as opposed to simply using them. They stated that catch-up and imitation could take place concurrently. When firms develop without sufficient capital and technology, they tend to use catch-up strategies. Perez and Soete [6] concluded that catch-up involves being in a position to take advantage of opportunities temporarily created by such technological transitions. Bell and Pavitt [7] considered that catch-up is not an automatic process but is based on accumulated and assimilated capabilities that necessitate learning. Closing the technology gap by forerunners mainly depends on the direction and rate of catch-up. Hung and Negassi [8] revealed that R&D dynamics caused by internal and external constraints obliged French firms to stay in the race to catch up and not be overtaken by their primary competitors. In East Asia, technological catch-up is typically involved in government institutions. Its adaptation and upgrading is visible, most conspicuously in South Korea and Taiwan [9].

#### 2.1.2. Leapfrogging

Leapfrogging is another strategy for achieving success when firms encounter fierce competition. Encaoua and Ulph [4] asserted that innovation takes the form of what can be called strong leapfrogging, in which the innovating firm must leapfrog the current insider to become a leader, necessarily making the innovator an outsider. Leapfrogging may be either weak or strong, depending on the technological gap. Weak leapfrogging is related to the situation in which the innovator obtains a transient leadership that can be contested by the laggard firm during the subsequent race. Strong leapfrogging occurs when the innovator obtains a monopoly on the product market because of a large technological gap. Davison et al. [10] showed that the specific use of IT to accelerate development and promote economic growth is often referred to as technology leapfrogging, which entails implementing a new and up-to-date technology in an application area in which at least the previous version of that technology has not been deployed. Consequently, leapfrogging may appear to be an attractive option for late adopters, but it may not always provide the intended results in all circumstances. Hoernig [11] indicated that leapfrogging in equilibrium occurs under Bertrand competition but may not occur under Cournot competition. (For more explanation in Bertrand and Cournot competition, please see Negassi and Hung [12].) Hoernig [11] revealed that traditional Schumpeterian leapfrogging models of drastic innovation are more appropriate if market competition is high, whereas the stepwise assumption is justified when competition is less intensive. Binz et al. [13] indicated three necessary conditions for a leapfrogging: first, a minimal endowment with a basic infrastructure as well as technological and organizational absorptive capacity; second, government interventions used to strengthen incentives for the uptake of innovation technologies in newly industrializing countries (NIC); and finally, technology transfer and financial assistance from developed economies.

Technological innovation can become a source of long-term productivity and economic growth when specific institutions are established to focus on the development of human capital, particularly technical and scientific skills. Li [14] stated that technological leapfrogging refers to the process and approach by which developing countries catch up with the leaping or linearity accelerative development of the technological contrail of technological advanced developing countries or find a separate new route to develop technology and economics rapidly. Upadhyaya and Singh [15] depicted using leapfrogging in a technical sense to signify skipping the technological frontier or product cycle. Huang [16] demonstrated that less developed countries have previously conducted leapfrogging in, for example, telecommunication technology, and therefore benefit more than other countries do. However, such beliefs regarding leapfrogging are not prevalent.

#### 2.1.3. Catch-Up or Leapfrogging?

Schumpeter's framework, the core of the “process of economic development,” is a virtuous interaction between finance and competition through innovation, which develops as a struggle for survival and growth in a structurally uncertain environment [17]. The profits that result from dominant market positions are always under threat from imitative strategies or other firms' innovative behaviors; these profits can be maintained only by continual productivity enhancement and product differentiation [18]. Schumpeterian competition of creative destruction is a permanent leapfrogging process in which forging ahead and falling behind are anticipated results [17]. Furthermore, no fixed technological “frontier” exists. Competition is a perpetually redefining and reinventing process. Catch-up is a relatively loose framework that is similar to the Rostovian concept (also called the Rostovian takeoff model) of a linear path of development toward an equilibrium imposed on history rather than a framework of continual, structural, and cumulative change and creative destruction [19]. A Schumpeterian approach, centered on the concept of leapfrogging through innovation, is outlined as a promising approach to addressing both development theory and historical trajectories.

Catch-up or leapfrogging accounts have been diversely dependent on industries and countries. Lee and Lim [20] reviewed South Korean industries and indicated that some industries have achieved remarkable catch-up or leapfrogging and continue to have positive prospects for the near future, whereas others are facing severe difficulties after a certain amount of catch-up. Lee and Lim [20] sought to extend the question of how catch-up is possible to the questions of what the generalized conditions are for successful catch-up and whether various catch-up patterns, such as leapfrogging, exist. Malerba and Orsenigo [21] classified road vehicles, engines, telecommunications, and semiconductors as belonging to the Schumpeter model. Finally, numerous studies have constructed the leapfrogging or catch-up strategy separately, but few studies have focused on the leapfrogging or catch-up strategy simultaneously.

### 2.2. Technological Race

A technological race is an interactive pattern characterized by firms that attempt to exceed their competitors or not fall too far behind, and the leapfrogging strategy is assumed to occur more frequently than the catch-up strategy does. Firms that are behind their race exhibit a robust tendency to expedite innovative efforts to catch up with leading firms [22]. Thus, a major focus of this approach is the strategic orientation of corporations in participating in such a race, revealing empirically observable phenomena such as catch-up and leapfrogging, as supported by statistical measurements. Therefore, the largest prize in a race is awarded to the first participant to cross a well-defined finish line. Similarly, the first firm to make an industrial breakthrough often captures the vast share of industry profits [23]. Gottinger [24] showed that a primary consideration of technological competition is the observation that firms are engaged in a race toward a larger market share. In a strong competition race, strategy plays a critical role. The participants adjust their strategy as the race develops, particularly in response to changing their relative positions.

A technological process is exclusively based on the experiences of each firm and measured according to the time that each firm is faithful to the research project [25, 26]. A firm that is primarily devoted to research thus has the largest technological capital. The only winner of the race is the first to reach a certain level of technological capital. Because there can be only one winner, one of the two firms will abandon the race, and the firm with less crucial experience, instead of the winner, will continue the race. Previous studies have recognized that the conclusions of the model—the equilibrium of *ε*-preemption—rest on the technological process [8]. A late-coming firm does not enter the race because it does not have the chance to catch up with its competitor. The impossibility of leapfrogging accurately comes from the technological process, and later models aim at practically modeling the technological process [4]. Encaoua and Ulph [4] employed numerous specifications to obtain realistic modeling of the technological process. These modifications thus provide a chance to the late-coming firm to catch up with its rival, only if it is sufficiently supported by its own incentives to innovate with its technological competence.

Market leaders continue investing in new technologies to maintain their leadership, whereas followers take risks to catch up with leaders. This often promotes technological leapfrogging [20]. A consequence of this technological competition is frequent changes. Hence, economists have extensively studied whether the follower or leader innovates more. It is suggested that modeling the innovation process according to an auction game implies that the firm with the largest incentives to innovate is the winner in the technological race [27, 28]. However, modeling a specific innovation according to an auction game is certainly simplistic, and several models have been proposed to reevaluate tenuous assumptions that have reduced the empirical significance of this modeling [8, 20]. This is because the empirical verifications of modeling innovation process remain fragmented and are often unsatisfactory [12].

In contrast to previous studies that have primarily used financial capacities or seller concentration (e.g., a bias could be caused by large firms with greater capacities that are more innovative; however, most Taiwanese firms are small and medium enterprises (SMEs)), this study incorporated the organization view (e.g., employee) and innovation strategy (e.g., patent) into the innovation process to investigate the competition strategy of firms. Furthermore, this study examined information on numerous databases by using a pooling method. The Second Taiwan Technological Innovation Survey (TTIS II) explored several technological innovation activities. Linked to the annual survey of manufactures and their patent data, the TTIS II facilitates high-level econometric analysis. This approach and its results are novel in certain ways. In particular, the pooling method proposed in this study is based on segmenting firms according to groups or industries rather than individual firms, which prompts using a greater number of general theories than those normally considered in typical panel models [29].

In summary, a primary consideration of technological competition is to acquire a larger market share or enhanced turnovers. Although previous studies have shown that modeling a specific innovation according to an auction game indicates that the firm with the largest incentives to innovate is the winner in the technological race, few studies have discussed “catch-up” or “leapfrogging” strategies. Therefore, this study investigated the advantage management strategies of a firm regarding the technological race in the manufacturing sector; that is, this study tried to reveal whether firms adopt a catch-up or leapfrogging strategy in the competition for innovation.

## 3. Methodology

### 3.1. Databases

This study linked five sources, namely, the TTIS II, Directorate-General of Budget, Accounting and Statistics (DGBAS) from Executive Yuan, R.O.C (Taiwan), Taiwan Industry Economics Services (TIES), patent database from the Taiwan Intellectual Property Office (TIPO), and the R&D survey from the Department of Statistics, Ministry of Economic Affairs from the 2003–2007 period. The research databases are shown in Table 1. The nomenclature of the Taiwanese manufacturing industry is classified into 12 categories, presented in Table 2 as 12 subindustries.

### 3.2. Model

This study classified variables into competition variables and innovation variables. The principal variable is the degree of competition. To analyze a firm's advantage management strategy, a system of three linear equations was developed as follows: (1) is used to determine the total system effect on competition, (2) is used to measure the total system effect on innovation, and (3) is used to measure the total system effect on turnovers:

(1)
Comp
=α0+α1RD_E+α2Mks +α3Cap+α4RD_T+α5Ler+α6Pat+εi

(2) Inno=β0+β1RD_E+β2Mks+β3Cap+β4RD_T +β5Ler+β6Tur+β7Dum

(3) Sa=γ0+γ1RD_E+γ2Mks+γ3Cap +γ4Tur+γ5Ler+γ6Pat+εi,

where Comp denotes the competition degree measured according to R&D expenditure, market share, capital, R&D turnovers, the Lerner index, and number of patents. Inno indicates the innovation degree measured according to R&D expenditure, market share, capital, R&D turnovers, the Lerner index, turnovers, dummy of time, and dummy of the technology sector. Sa signifies the increasing or decreasing degree of a firm's turnovers measured according to R&D expenditure, market share, capital, turnovers, the Lerner index, and number of patents. RD_E refers to R&D expenditure, Mks represents the market share, Cap means capital, RD_T refers to R&D turnovers, Ler is the Lerner index, Pat represents the number of patents, Tur indicates turnovers, and Dum refers to the dummy of time and the dummy of the technology sector. *α*_0_, *β*_0_, and *γ*_0_ represent the intercept. *α*_1_, *α*_2_, *α*_3_, *α*_4_, *α*_5_, *α*_6_, *β*_1_, *β*_2_, *β*_3_, *β*_4_, *β*_5_, *β*_6_, *β*_7_, *γ*_1_, *γ*_2_, *γ*_3_, *γ*_4_, *γ*_5_, and *γ*_6_ are the coefficients of measured variables. Finally, *εi* is the residual.

This study used simultaneous equation econometrics to determine advantage strategies in the Taiwanese manufacturing industry. However, a more practical justification of this study is that traditional estimation methods developed for a single equation can no longer be directly applied. The simultaneous equation estimation systems are excellent in most respects because of the omission of disturbing variables. Epple and McCallum [30] used simultaneous equation econometrics systems to explain the vehicle industry, and the systems approach produces an estimated supply function in which quantity produced is an increasing and statistically significant function of price.

### 3.3. Measuring Variables

#### 3.3.1. R&D Expenditure

R&D expenditure is crucial for company growth, and companies are typically willing to invest in R&D research. Grossman and Shapiro [23] indicated that leaders engage in R&D more intensively than followers do and that both types of firm intensify their efforts if the follower catches up with the leader. Therefore, R&D expenditure is an index of innovation and competition, an investment that depends on the increase of capital stock and a percentage of R&D intensity [23], as well as a proxy for innovation and competition. The design and development of new products derive from specific R&D activity. In this study, R&D expenditure was a crucial variable for measuring the advantage strategy model. Among numerous studies, Lee and Wilde [31] and Reinganum [32] have investigated the determinants of R&D expenditures in situations in which the first to succeed captures the largest (or only) prize. Their analyses embodied no notion of progress or leadership; however, they studied stationary races in which all firms are equally placed until the competition is (suddenly) over. Grossman and Shapiro [23] indicated that the leader always devotes more resources to R&D expenditure than does the follower, but if the follower catches up, both firms intensify their efforts. In addition, the numerical simulations for a wide range of parameter values suggest that in a typical pattern, the leading firm increases its R&D expenditures when it advances to the final phase, whereas the follower reduces the extent of its research activity. Therefore, R&D expenditure was a critical variable in the current study.

#### 3.3.2. R&D Turnovers

R&D turnovers are incomes from sales of R&D goods or sales of the R&D process. Few researchers have used this variable to detect market competition. Yen and Chang [33] observed that R&D turnovers can pinpoint the effect of R&D investment in the Taiwanese manufacturing industry. The current study used R&D turnovers to calculate competition levels and multiplied the number of R&D turnovers by the R&D turnover ratio. All of the turnovers are from TIES; the R&D turnovers ratio is from the Department of Statistics, Ministry of Economic Affairs. Finally, R&D turnovers were a crucial factor in this study, because of being a critical variable in the advantage strategy model in the Taiwanese manufacturing industry.

#### 3.3.3. Turnovers

Turnovers are incomes from sales of goods or services. Researchers have adopted this variable to calculate other numbers, such as R&D turnovers, the Lerner index, and market share. Li and Vanhaverbeke [34] computed market share according to the sales amount, which they applied to detect R&D returns. Tingvall and Poldahl [35] calculated the HHI according to the sales amount, and numerous researchers have also used the HHI or the Lerner index to calculate market intensity. The number of turnovers in the descriptive statistics indicates how much money is earned in the manufacturing industry. Therefore, turnover was a crucial variable in this study.

#### 3.3.4. Capital

Capital is an investment similar to R&D investment [23]. These two types of investment interpenetrate firms' strategies to overcome the markets. Producing new goods from R&D typically implies installing new physical-production capacities. Capital can be used as a variable to measure the size and financial state of a firm. Thus, we used capital to determine the relationship between turnovers and firm size. Descriptive statistics clarify the relationship among capital, turnovers, and R&D expenditure. Some firms have more capital and invest further in R&D to obtain additional turnovers. Capital was regarded as a crucial factor in the Taiwanese manufacturing industry in this study.

#### 3.3.5. Patent

Gilbert and Newbery [25] indicated that leadership is difficult to retain when technological opportunities exist. Therefore, we measured technological opportunities based on the total number of granted patents in a particular sector. A patent is considered extremely effective in the juridical protection of innovations [36]. The current study used patent as a variable based on its representation of innovation and investigated the relationship between the number of patents and R&D expenditure. Previous studies on technological innovations have generally used patent citation data as a proxy of innovation between firms and industries.

R&D rivalry represents a single innovation. More than one research team can pursue a new technology, and intellectual property rights over the innovation are potentially weak. Hence, numerous enterprises have invested additional funds into R&D and have protected their innovation by using patents. However, new-product design and development derive from R&D, which is the source of technological progress for a firm and plays an essential role in innovation as the only resource of abundant and liable statistics. R&D expenditure is an investment that depends on the increase of capital stock [23] and accounts for a specific percentage of R&D intensity [23]. For instance, the rate at which R&D is translated into successful innovations varies among industries and firms. In addition, several firms may engage in the same R&D concurrently, generating redundant R&D expenditures. However, some innovation incurs few or no expenditures, or at least not in the context of accounting. Similar to applying patent data, using R&D expenditures or R&D intensity as indicators of innovation may result in either overestimates or underestimates. Thus, we used the number of patents based on the TIPO in the Taiwanese manufacturing industry to measure the degree of innovation. The nonmetal mineral industry has no data in the TIPO. Finally, patents constituted a crucial variable used to measure innovation degree in this study.

#### 3.3.6. Competition

Market share and the Lerner index are two main indicators for measuring the degree of market competition. This study applied market share and the Lerner index [37] as competition indicators. The Lerner index function is (*P*−MC)/*P*, where *P* is the price and MC is the marginal cost. However, the Lerner index is regarded as a mathematics inference; MC is highly theoretical and unobserved [38]. The Lerner index can be extended to (*P*−MC)∗*Q*/*P*∗*Q*, where *Q* is the quantity. This extended form is the ratio of the gross profit and sales amount [12].

Market share was also used to measure competition and can be used to measure market power and business performance. Market share is the ratio of the sales amount of an enterprise to that of its sector. This study summarized the sales amount based on various industry levels and calculated the market ratio of different industries.

#### 3.3.7. Employee

An employee contributes labor and expertise to perform specific duties. In most modern economies, the term “employee” refers to a specifically defined relationship between an individual and a corporation, which differs from that between a customer and a client. Empirical literature on the effects of innovation on employment has significantly progressed since the 1990s, when microeconomic data on individual firms became widely available. Brouwer et al. [39] observed a positive effect on product innovation and employment growth but a negative effect on overall innovation. Perez and Soete [6] showed a significantly positive effect of product innovation on employment. Manpower improved performance because of increased labor productivity and improvements in managerial functioning. Archibugi et al. [40] demonstrated that employees can be used as a measure of firm size and innovation cost [8]. Moreover, labor productivity is the basis for manufacturing growth. In summary, this study used the employee as an instrumental variable to measure firm size and function.

#### 3.3.8. Gross Profit

In accounting, gross profit is the difference between revenue and the cost of manufacturing a product or providing a service, before overhead, payroll, taxation, and interest payments are deducted. Gross profit can be determined by deducting the cost of goods sold and is equal to net sales after the cost of goods sold is subtracted. However, gross profit should not be confused with net income, which is equal to gross profit after total operating expenses, taxes, and interest are subtracted. The competition degree is measured according to the market share and Lerner index, which are calculated based on gross profit [8]. Researchers have adopted this variable to detect market competition. The current study used gross profit as an instrumental variable to calculate market share and the Lerner index.

#### 3.3.9. Dummy of the Technology Sector

Hung and Negassi [8] recently demonstrated that the technology sector is composed of two sectors, high technology and low technology. The dummy of the technology sector classifies industries into high and low technologies. A dummy equal to 1 indicates that the industry is high technology, whereas a dummy equal to 0 demonstrates that the industry is low technology. Li and Vanhaverbeke [34] used a unique dataset based on 1,021 firms in several countries, observing that a firm's response to competitive market pressure depends on its level of technological competence: firms with a high level of technological competence increase their R&D effort, whereas firms with low technological competence reduce it. Researchers have shown that late entrants overtake pioneers in various markets, including high-tech industries such as personal computers and cameras, as well as low-tech categories such as food processors, ballpoint pens, and light beer [41]. In the current research, the general petrochemical industry, basic metal processes industry, mechanical electronic and transportation industry, and fuel industry represent high technology; other industries demonstrate low technology.

#### 3.3.10. Dummy of Time

The dummy of time represents a time-trend variable. A dummy equal to 0 refers to the same period; when it equals 1, it refers to a different period. Hung and Negassi [8] indicated that the dummy of time is a crucial factor of the model. The dummy of time is used to measure the time lag from 1992 to 2004 in the manufacturing industry. In this study, the dummy of time was a time-effect indicator.  Table 3 lists the justified variables constructed in this study.

### 3.4. Justification of Methods

#### 3.4.1. Panel Regression

This study used the industry level for panel data, combining cross-sectional and time-series data, models, and the fixed effect model for estimation. The two types of panel-data regression model are the (a) fixed-effect model and the (b) random-effect model. The Hausman test differentiates between the random-effect model and the fixed-effect model. The hypothesis is as follows:

 
*H*_0_: *E*(*u*_*it*_∣*X*_*it*_) = 0,
 
*H*_1_: *E*(*u*_*it*_∣*X*_*it*_) ≠ 0.

When *u*_*it*_ = *u*_*i*_ + *v*_*it*_ under the null hypothesis, the random-effect model is more suitable; when the test rejects the null hypothesis, the fixed-effect model is more appropriate.

#### 3.4.2. Two-Stage Least Squares Regression

Two-stage least squares (2SLS) regression ensures the coefficient estimates of bias and consistency. This study used the 2SLS regression model to explore the effect of R&D expenditure on enterprise advantage strategies. The first stage of 2SLS regression is structural form, which involves using ordinary least squares (OLS) to obtain parameters and endogenous variables; the second stage entails using each endogenous variable in the structural form and subsequently using OLS regression to obtain the parameter estimates of structural form.

#### 3.4.3. Tobit Regression

The Tobit model, also called a censored regression model, is designed to estimate the linear relationships among variables when either left censoring or right censoring exists in the dependent variable (also known as censoring from below and above, resp.). Censoring from above is performed when all cases with a value at or above a certain threshold assume the threshold value, so that the true value might be equal to the threshold but may also be higher. In the case of censoring from below, values that fall at or below a certain threshold are censored. Using the least squares method to evaluate this model led to biased estimates. Adopting the Tobit regression model helped reduce this problem.

## 4. Results and Discussion

### 4.1. Descriptive Statistics

Table 4 lists the descriptive statistics of the variables. The market share, Lerner index, R&D expenditure, R&D turnovers, and patents are the main variables in the study.

Table 5 lists the coefficient correlations. The Lerner index and market shares as a measure of leadership strategy and financial constraints have a different focus, which is partially supported by the small correlation coefficient among the indicators.

Table 4 shows that the degree of competition (market share and the Lerner index) in the Taiwanese manufacturing industry is competitive, which implies that Taiwanese manufacturing firms tend to catch up with their rivals. This study also observed that Taiwanese manufacturing firms in the high-technology industry encounter fiercer competition than do those in the low technology industry. Taiwanese manufacturers primarily focus on 3C (computer, communication, and consumer electronics) commodities. The aforementioned result can easily explain why the product life cycle of 3C commodities is extremely short and why maintaining or pursuing a higher market share is difficult (Table 4).

In addition, Taiwanese manufacturers tend to invest research funds in R&D. This study found that R&D expenditure in the high-technology industry is higher than that in the low technology industry (the minimum R&D expenditure in Taiwan is 1,000 New Taiwan Dollars (NTD), the maximum is 4,333,782,000 NTD, and the mean is 319,627,270 NTD) (Table 4). The statistics regarding R&D turnovers indicate that the Taiwanese manufacturing industry has a low profit rate compared with R&D expenditure (4.88%) (Table 4). The result reveals that although numerous Taiwanese manufacturers belong to the high-technology industry, the majority are original equipment manufacturers. PC manufacturing firms have yielded low profit, even lower than 1% or 2% in previous years.

The high-technology industry has abundantly more patents than the other industries in this study do; however, when considering the entire Taiwanese manufacturing industry, the number of patents is not high. This result also indicates that R&D input (R&D expenditure) could turn to R&D output (patent) primarily in the high-technology industry, but this output does not effectively “translate into” or “protect” profit.

### 4.2. Regression Results

Table 6 lists the results from using the panel, 2SLS, and Tobit regressions. The main findings show that the influence of firms facing competition in the system (i.e., the Taiwanese manufacturing industry in this study) is extremely intensive, the system innovation effect is moderate, and the system yields different results from total and R&D turnovers. The variables measured for the advantage strategy, R&D_E(−1), R&D_T(−2), Mks(−3), Ler(−3), and Pat(−3), are statistically significant at 1% or 10% level (Table 6).

Therefore, the results indicate that (i) the competition is fierce in the Taiwanese manufacturing industry and (ii) Taiwanese manufacturers attempt to catch up with their competitors to remain in the technological race. This is because the majority of the Taiwanese firms are SMEs, and they do not respond quickly to competition (although they have been traditionally regarded as flexible and quick to respond). Moreover, the results of R&D expenditure (R&D_E(−1)) and R&D turnovers (R&D_T(−2)) explain that firms willing to invest more in R&D activities will achieve more R&D turnovers; when firms invest more in R&D expenditure, competition becomes intensive. When firms achieve more R&D turnovers, they tend to invest more in R&D expenditure. This result could indicate that, under financial constraints, Taiwanese manufacturing firms attempt to invest in R&D to catch up with their rivals or to avoid being eliminated from the race.

The result also suggests that only the patent (Pat(−3)) is negative. This confirms the discussion on the descriptive statistics. The innovation effect does not play a crucial role. Except for firms in the high-technology industry (the dummy of the technology sector is positively significant), Taiwanese manufacturers invest more in R&D expenditure or achieve more R&D turnovers, but they do not expend much effort into patenting, or perhaps the patent quality is poor. If they violate the patents of their rivals, they will be required to pay a fine or buy the patent rather than create more patents. Not all patents are crucial or profitable. Hence, companies that are willing to invest more funds in R&D expenditure are unwilling to spend on patents.

## 5. Conclusion

The technological competition is an interactive pattern characterized by firms constantly trying to move ahead of their rivals or trying not to fall too far behind. Numerous authors have indicated that being in the lead entails disproportionately large payoffs. In the competition for innovation, firms continually increase innovation to raise their competitive power. The primary consideration of technological competition is to gain a larger market share or enhanced turnovers, which firms can achieve through “catch-up” or “leapfrogging” strategies. However, regarding the technological race, few studies have discussed these strategies. This study investigated the advantage management strategies of a firm via the technological race in the manufacturing sector. The results indicated that the “catch-up” strategy would occur more frequently than the “leapfrogging” strategy does. In addition, Taiwanese manufacturing firms (mostly SMEs) in intensive competition tend to adopt the “catch-up” strategy to keep up with their competitors in order to remain in the technological race. The results also explain that firms willing to invest more in R&D activities will achieve more R&D turnovers; when firms invest more in R&D expenditure, competition becomes intensive. They attempt to invest in R&D, but the result is ineffective because they do not expend much effort into patenting, or the patent quality is poor; hence, they cannot protect their innovation. Finally, the robust results of this study verify the approach of technological race, which reduces tedious assumptions in auction game, applied to the process of research in the Taiwanese manufacturing industry.

## Figures and Tables

**Table 1:** Research databases.

Databases	Data	Period	Number of firms
TTIS II (Second Taiwan Technological Innovation Survey)	Manufacturing Industry Classification	2003–2007	10,000

DGBAS (Directorate-General of Budget, Accounting and Statistics, Executive Yuan, R.O.C (Taiwan))	Manufacturing Industry Classification	2003–2007	—^†^

Department of Statistics, Ministry of Economic Affairs	R&D turnovers ratio	2003–2007	142,500

TIPO (Taiwan Intellectual Property Office)	Patent	2003–2007	40,000

TIES (Taiwan Industry Economics Services)	Industry	2003–2007	640,000

Source: TTIS II, TIPO, TIES, Directorate-General of Budget, Accounting and Statistics (DGBAS) from Executive Yuan, R.O.C (Taiwan), and Department of Statistics, Ministry of Economic Affairs.

^†^DGBAS is used only for the classification of the Taiwanese manufacturing industry.

**Table 2:** 12 subindustries of the Taiwanese manufacturing industry.

Industry		Industry	
T1	Food and tobacco industry	T7	Metal products industry
T2	Textile industry	T8	Mechanical electronic and transportation industry
T3	Paper-making industry	T9	Furniture industry
T4	General petrochemical industry	T10	Fuel industry
T5	Nonmetal mineral industry	T11	Waste water industry
T6	Basic metal processes industry	T12	Construction industry

**Table 3:** Justification of variables.

Variables	Source	Justification of variables
R&D expenditure (RD_E)	Taiwan Industry Economics Services (TIES)	This study uses R&D expenditure as a proxy for innovation.

R&D turnovers (RD_T)	Taiwan Industry Economics Services (TIES)	The study uses R&D turnovers to calculate the competition level.

Turnovers (Tur)	Taiwan Industry Economics Services (TIES)	This study uses turnovers to calculate the Market Share and Lerner index.

Capital (Cap)	Taiwan Industry Economics Services (TIES)	Size of firms

Patent (Pat)	Taiwan Industrial Property Office (TIPO)	The relationship between number of patents and R&D expenditure

Competition (Comp)	Taiwan Industry Economics Services (TIES)	Market share and the Lerner index are two main indicators to measure market. The study chooses it to calculate in the real world

Employee (Emp)	Taiwan Industry Economics Services (TIES)	This study uses employee to measure the firm size

Gross profit (GP)	Taiwan Industry Economics Services (TIES)	Market share and Lerner index

**Table 4:** Descriptive statistics.

	Number of firms	Minimum	Maximum	Mean	Std. deviations
R&D expenditure∗	10,018	1	4,333,782	319,627.27	1,027,852.96
R&D turnovers∗	10,018	955.888	211,308.965	40,971.85	61,661.97
Turnovers∗	10,018	19,828	4,383,185	849,879.74	1,279,055.20
Capital∗	10,018	5,038	4,424,744	451,546.53	943,329.58
Patent	10,018	0	24,321	3,479.64	6,155.42
Market share	10,018	0.001829	0.49215282	0.09	0.12
Lerner index	10,018	0.024732	0.33590200	0.16	0.59
Employee	10,018	13	294	78.57	80.60
Gross profit	10,018	3,444	1,029,373	153,739.04	257,984.51

*Unit: NTD$.

**Table 5:** Correlation matrix.

	RD_E	Mks	Cap	RD_T	Ler	Pat	Tur	GP	Emp
RD_E	1								
Mks	0.197	1							
Cap	0.141	0.850	1						
RD_T	0.188	0.952	0.784	1					
Ler	0.066	0.320	0.474	0.224	1				
Pat	−0.125	0.410	−0.176	−0.387	−0.059	1			
Tur	0.188	0.952	0.784	1.000	0.224	−0.387	1		
GP	0.170	0.949	0.941	0.934	0.408	−0.287	0.934	1	
Emp	0.142	0.623	0.223	0.606	0.017	0.802	0.606	0.434	1

**Table 6:** Simultaneous equations econometrics approach.

	Panel	2SLS	Tobit
Constant	1330.8 (0.0021)∗∗∗	1395.5 (0.0036)∗∗∗	1395.6 (0.0095)∗∗∗
R&D_E (−1)^†^	0.34403 (0.0000)∗∗∗	0.35038 (0.0000)∗∗∗	0.34304 (0.0099)∗∗∗
Mks (−3)	1827.73 (0.0098)∗∗∗	1871.58 (0.0096)∗∗∗	1871.58 (0.0099)∗∗∗
Cap (−3)	0.02334 (0.0893)∗	0.01496 (0.0936)∗	0.00110 (0.0999)∗
R&D_T (−2)	1.57519 (0.0052)∗∗∗	1.37347 (0.0062)∗∗∗	1.10365 (0.0097)∗∗∗
Ler (−3)	1512.7 (0.0935)∗	1565.2 (0.0833)∗	1565.1 (0.0991)∗
Pat (−3)	−2.01825 (0.0588)∗	−1.93092 (0.0902)∗	0.77388 (0.0853)∗
Tur (−2)	0.15110 (0.0892)∗	0.16098 (0.0230)∗∗	0.12511 (0.0998)∗
Dummy time	0.13415 (0.0208)∗∗	0.76467 (0.0845)∗	0.76467 (0.0974)∗
Dummy of the technology sector	0.13718 (0.0605)∗	0.72172 (0.0867)∗	0.72172 (0.0998)∗
Adj *R*-squared	0.769571	0.770088	—

Notes: *P* values within brackets ∗∗∗, ∗∗, and ∗ indicate significance at the 1%, 5%, and 10% level, respectively. ^†^(−*n*) represents *n* year(s) of time lag.
